# The correlation between the number of eligible patients in routine clinical practice and the low recruitment level in clinical trials: a retrospective study using electronic medical records

**DOI:** 10.1186/1745-6215-14-426

**Published:** 2013-12-11

**Authors:** Eriko Sumi, Satoshi Teramukai, Keiichi Yamamoto, Motohiko Satoh, Kenya Yamanaka, Masayuki Yokode

**Affiliations:** 1 Department of Clinical Innovative Medicine, Institute for Advancement of Clinical and Translational Science, Kyoto University Hospital, 54 Shogoin Kawahara-cho, Sakyo-ku, Kyoto 606-8507, Japan; 2 Innovative Clinical Research Center, Kanazawa University, Kanazawa, Japan; 3 Division of Clinical and Epidemiological Database, Department of Information Governance, National Cerebral and Cardiovascular Center, Osaka, Japan; 4 Department of Surgery, Graduate School of Medicine, Kyoto University, Kyoto, Japan

**Keywords:** Clinical trials, Research patient recruitment, Eligibility determination, Clinical informatics, Accrual

## Abstract

**Background:**

A number of clinical trials have encountered difficulties enrolling a sufficient number of patients upon initiating the trial. Recently, many screening systems that search clinical data warehouses for patients who are eligible for clinical trials have been developed. We aimed to estimate the number of eligible patients using routine electronic medical records (EMRs) and to predict the difficulty of enrolling sufficient patients prior to beginning a trial.

**Methods:**

Investigator-initiated clinical trials that were conducted at Kyoto University Hospital between July 2004 and January 2011 were included in this study. We searched the EMRs for eligible patients and calculated the eligible EMR patient index by dividing the number of eligible patients in the EMRs by the target sample size. Additionally, we divided the trial eligibility criteria into corresponding data elements in the EMRs to evaluate the completeness of mapping clinical manifestation in trial eligibility criteria into structured data elements in the EMRs. We evaluated the correlation between the index and the accrual achievement with Spearman's rank correlation coefficient.

**Results:**

Thirteen of 19 trials did not achieve their original target sample size. Overall, 55% of the trial eligibility criteria were mapped into data elements in EMRs. The accrual achievement demonstrated a significant positive correlation with the eligible EMR patient index (r = 0.67, 95% confidence interval (CI), 0.42 to 0.92). The receiver operating characteristic analysis revealed an eligible EMR patient index cut-off value of 1.7, with a sensitivity of 69.2% and a specificity of 100.0%.

**Conclusions:**

Our study suggests that the eligible EMR patient index remains exploratory but could be a useful component of the feasibility study when planning a clinical trial. Establishing a step to check whether there are likely to be a sufficient number of eligible patients enables sponsors and investigators to concentrate their resources and efforts on more achievable trials.

## Background

Clinical trials are essential for gaining and extending knowledge about new therapies, and sufficient patient enrollment in clinical trials is critical to fulfill their scientific objectives. Nevertheless, a number of trials have failed to achieve their target sample size within the original accrual period [1-3]. For such trials, extending the accrual period, modifying the eligibility criteria or, in the worst case scenario, prematurely closing the trial may be necessary. Moreover, many investigators continue to make the same mistakes despite the great advances made in handling clinical trials data using information technology [4].

Many reasons for the low levels of recruitment have been cited, including fewer eligible patients than expected, a smaller percentage of patients agreeing to participate [1,5], time constraints, resource issues, consent interviews and difficulties in identifying the patients [6].

Recently, many screening systems that search electronic medical records (EMRs) or clinical data warehouses derived from EMRs for patients eligible for clinical trials have been developed, and their efficiencies have been evaluated [7-9]. The number of patients who meet the eligibility criteria when medical records are manually reviewed is less (13 to 74%) than the number of potential trial patients identified by an electronic screening system [7,10-15]. Nevertheless, screening systems are promising in that they can provide information on the total eligible patient population at the planning stage of a clinical trial. Estimates of the number of eligible patients enable both the sponsors and the investigators to concentrate their resources and efforts on more achievable and conclusive trials. Moreover, investigators should not put their patients at risk by enrolling them in an inconclusive trial. For more reasonable research programs, we hypothesized that researchers can predict the difficulty of trial patient enrollment by estimating the number of eligible patients using EMR data. We explored how to estimate the number of eligible patients using the EMRs, and using a retrospective design, we tested our hypothesis that the number of eligible patients identified from EMRs correlates with the number of patients actually enrolling in clinical trials.

## Methods

### Trial data collection

The trials were identified using a departmental database from the Institute for Advancement of Clinical and Translational Science and the University Hospital Medical Information Network Clinical Trial Registry (UMIN-CTR) [16]. The investigator-initiated therapeutic trials that started between July 2004 and January 2011 were included if the following data were available: the trial eligibility criteria, target sample size, number of enrolled patients and accrual period at the Kyoto University Hospital (KUH). The trial eligibility criteria, number of scheduled and enrolled patients and the duration of enrollment were extracted from published papers or from registered information in the UMIN-CTR. For unpublished data, trial protocols and management lists in the departmental database were used after obtaining consent from the relevant principal investigators. The study protocol was approved by the Ethical Committee of the Graduate School of Medicine, Kyoto University (E1175).

### Electronic medical records retrieval system

We used an EMR retrieval system that was developed at the Institute for Advancement of Clinical and Translational Science to screen EMRs for patients in KUH [17]. In this system, EMR data, including diagnoses, medications and injections, laboratory tests, radiological or pathological studies, and operative notes, were extracted from the data warehouse to enable the comprehensive and efficient retrieval of patient data.

### The replacement of trial eligibility criteria with patient characteristics for a comparison to electronic medical records

We replaced the trial eligibility criteria of the trial protocol with patient characteristics that could be easily compared with EMR data and matched the translated criteria with the data elements in EMRs, referencing the methods of previous studies [10,11,17-21].

After the trial eligibility criteria were collected, three physicians discussed and replaced concepts in the eligibility criteria of each trial with patient characteristics, which were represented by codes, fixed terms or numeric data. Some medical concepts, such as ‘severe heart disease’, may be interpreted differently depending on the trial and the clinician caring for the patients. The three physicians discussed these concepts and made a general list of how to interpret these concepts as patient characteristics, such as considering a particular concept to be part of a group of diagnoses. The list also included instructions on how to replace the specific medical conditions that do not directly indicate one or more data elements in the EMRs by the data elements in the EMRs related to the conditions. For instance, we replaced the criterion ‘patients who do not need intravenous hyperalimentation’ with ‘no order for high-calorie infusion’. Namely, we aimed to estimate the number of patients who were already receiving care from the trial treatment or from alternative treatments in routine clinical practice rather than estimating the potential number of eligible patients still at the diagnostic stage. Concurrently, we re-categorized the eligibility criteria of the trial protocol into three categories: ‘Select’, ‘Omit’ and ‘Not applicable’. The ‘Select’ category indicates that the patient was included if he or she fulfilled the condition. The ‘Omit’ category indicates that the patient was excluded if he or she fulfilled the condition but was not excluded if the data were not available or were missing. The ‘Not applicable’ category indicates that the criterion cannot be searched in the EMRs because of missing or incomplete EMR data; for instance, the data were entered in plaintext freely, were captured as an image or were not entered. Thus, the items in the ‘Not applicable’ category were neither translated into computable eligibility criteria nor searched for in the EMRs.

Laboratory test requirements were indicated in either the inclusion or exclusion items of the trial eligibility criteria. If the laboratory tests were routinely performed, the requirements of the laboratory tests were categorized as 'Select', and if the test result fulfilled the requirement at least once, the patients were deemed eligible. If the tests were not performed routinely, the requirements of the laboratory tests were categorized as 'Omit'.

The periods of the order or the records to search were critical for estimating the number of patients. We searched for eligible patients using a primary criterion that was recorded during the year for which we wanted to know the number of eligible patients. For the other criteria, such as acute illness or diseases with no prior therapy, we searched eligible patients with other criteria in addition to the primary criterion recorded during the preceding two years for acute illness or diseases with no prior therapy and the previous five years for chronic or recurrent disease.

### The degree of concordance with the electronic medical record data

We examined how many and what type of trial eligibility criteria were mapped into the patient characteristics and corresponding data elements of the EMRs to evaluate the completeness of our mapping. We assigned patient characteristics, as mentioned above, to one of the 27 semantic categories defined by Luo *et al*. [22]. One author, a medical doctor, broke up and assigned the eligibility criteria to one of the semantic categories, and another medical doctor validated the results. Then, we counted the number of patient characteristics in the ‘Select’ or ‘Omit’ and ‘Not applicable’ categories.

### The number of eligible patients in the electronic medical records

We searched for potentially eligible patients using the EMR retrieval system. Each query in the EMR retrieval system was tested to find errors both in the program and in the search results. A system engineer then examined the number of patients in the ‘Select’ category, the ‘Select’ but not the ‘Omit’ category and the ‘Select’ and ‘Omit’ categories to confirm that the first number was equal to the sum of the second and third numbers. After this test, he obtained the estimated number of potentially eligible patients both in the year preceding the start of a trial and in the year in which a trial started using the EMR retrieval system. We did not perform an additional manual review of the medical records after the data extraction.

### Statistical analysis

The target sample size, number of patients actually enrolled, scheduled accrual period and actual accrual period were obtained.

The following formula was used to determine the eligible EMR patient index and the accrual achievement:

The eligible EMR patient index = the number of eligible patients identified by the EMRs per year/the target sample size per year.

The accrual achievement = the number of enrolled patients per year/the target sample size per year.

Where, the target sample size per year = the target sample size at KUH/the scheduled accrual period (year).

The number of enrolled patients per year = the number of patients actually enrolled at KUH/the actual accrual period (year).

We examined the relationship between the eligible EMR patient index and the accrual achievement using Spearman's rank correlation coefficient. The receiver operating characteristic (ROC) curves were used to determine the cut-off value for the eligible EMR patient index that identified the low enrollment trials with accrual achievements <1.0. Furthermore, we examined the consistency between the numbers of searched eligible patients in the year preceding the start of a trial and in the year in which a trial began to evaluate the reliability of the eligible EMR patient index. All statistical analyses were performed using R-2.14.1 and SAS software for Windows.

## Results

### Trial information

Of the 24 trials screened, 15 trials in the Institute for Advancement of Clinical and Translational Science database met the inclusion criteria, in addition to four trials in the UMIN-CTR (accessed on May 2, 2011). Table 1 indicates the characteristics of the 19 trials. The patient accrual period was extended in seven trials, and six trials recruited 100% or more of their target sample size within the scheduled accrual period.

**Table 1:** Characteristics of the trials

**Characteristic**	**Number of trials**
Phase	
I/II	7
II	6
IV	1
Not specified	5
Clinical areas	
Cancer	6
Internal medicine	6
Orthopedics	5
Others	2
Participating centers	
KUH only	17
Multicenter study	2
Study start date	
2004 to 2005	3
2006 to 2007	10
2008 to 2009	3
2010	3
Target sample size per year per center^a^	
0 to 9	6
10 to 19	6
20 to 29	2
30 to 39	1
≥40	4
Enrolled patients per year per center	
0 to 9	10
10 to 19	3
20 to 29	1
30 to 39	4
≥40	1

^a^The target sample size was calculated as the total sample size divided by the number of centers in a single trial for which an assigned sample size was not determined. KUH, Kyoto University Hospital.

### The replacement of trial eligibility criteria with patient characteristics for comparisons with the electronic medical records

**Figure 1:**
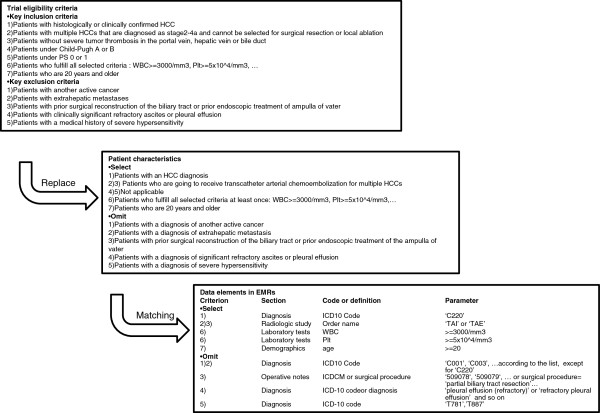
**An example of replacement of trial eligibility criteria with patient characteristics for comparisons with the electronic medical records (EMRs).** The trial eligibility criteria, replaced patient characteristics and matched data elements of EMRs are presented in the first, second and third columns, respectively. HCC, hepatocellular carcinoma; PS, performance status; TAE, transcatheter arterial embolization; TAI, transcatheter arterial infusion chemotherapy.

We replaced and matched the trial eligibility criteria for the 19 trials with the data elements in the EMRs. We present an example of this process in a trial titled ‘A randomized controlled study of the effectiveness of transcatheter arterial chemoembolization with cisplatin and transcatheter arterial chemoembolization with epirubicin for multiple hepatocellular carcinomas’ (Figure 1). The trial candidates were patients who were to receive transcatheter arterial chemoembolization for multiple HCCs at stage 2 to 4a without thrombosis in the portal vein, hepatic vein and the bile duct, although the trial eligibility criteria were only presented as the disease conditions. Therefore, we replaced the disease condition with the related standard treatment for the patients and assessed the radiologic study order in EMRs to narrow down the trial candidates.

### The degree of matching with the electronic medical record data

The 318 eligibility criteria from the 19 trials were transformed into 425 patient characteristics. Of the 425 patient characteristics, 408 were related to 18 semantic categories, and 17 were related to a ‘ no fitting category’. We found that 55% (235 of 425) of the characteristics in the eligibility criteria were matched with data elements in the EMRs. Compared with a previous study by Kopcke *et al*. [21], the degree of matching was similar with respect to both the total and the category (Table 2). The degree of match for each trial ranged from 38% to 75% (median 54%), and the degree was 50% in three trials.

**Table 2:** Degree of translation to the electronic medical record (EMR) data

	**Patient characteristics**	**Data elements in EMRs**	**Degree of translation**^**a**^	**Previous study**^**b**^
Health status	236	141	0.61	0.60
Disease, symptoms and signs	120	80	0.68	0.81
Pregnancy-related activity	12	0	0	0.16
Neoplasm status	24	16	0.67	0.75
Disease stage	10	1	0.10	0.25
Allergy	12	4	0.33	0.17
Organ or tissue status	54	40	0.75	0.74
Life expectancy	4	0	0	0
Treatment or healthcare	45	24	0.55	0.57
Pharmaceutical substance or drug	26	10	0.40	0.35
Therapy or surgery	19	14	0.74	0.74
Device	0	0	NA	0
Diagnostic or lab results	84	47	0.56	0.54
Diagnostic or lab results	84	47	0.56	0.54
Receptor status	0	0	NA	0
Demographics	21	21	1.00	0.85
Age	20	20	1.00	0.95
Special patient characteristic	0	0	NA	0.33
Literacy	0	0	NA	0
Gender	1	1	1.00	1.00
Address	0	0	NA	0
Ethnicity	0	0	NA	0
Ethical consideration	12	0	0	0.08
Consent	8	0	0	0.06
Enrollment in other studies	1	0	0	0
Capacity	2	0	0	0.16
Patient preference	1	0	0	0
Compliance with protocol	0	0	NA	0
Lifestyle choice	10		0.20	0.82
Addictive behavior	5	0	0	0.90
Bedtime	0	0	NA	0
Exercise	0	0	NA	0
Diet	5	2	0.40	0
No fitting category	17	0	0	-
Total	425	235	0.55	0.55

^a^The degree of translation = the number of patient characteristics/the number of data elements in EMRs.

^b^Previous study: the fraction of documentable patient characteristics in previous study [21]. The authors calculated the fraction of patients with any data in at least one corresponding data element for each patient characteristic.

### Data retrieval, correlation and receiver operating characteristic analysis

**Figure 2:**
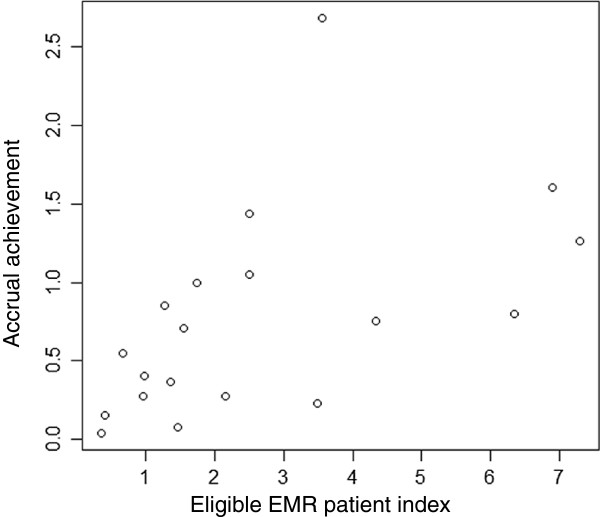
**Correlation between the eligible electronic medical record (EMR) patient index and the accrual achievement in 19 trials.** The eligible EMR patient index = the number of eligible patients identified from the EMRs per year/target sample size per year. The accrual achievement = the number of enrolled patients per year/target sample size per year.

**Figure 3:**
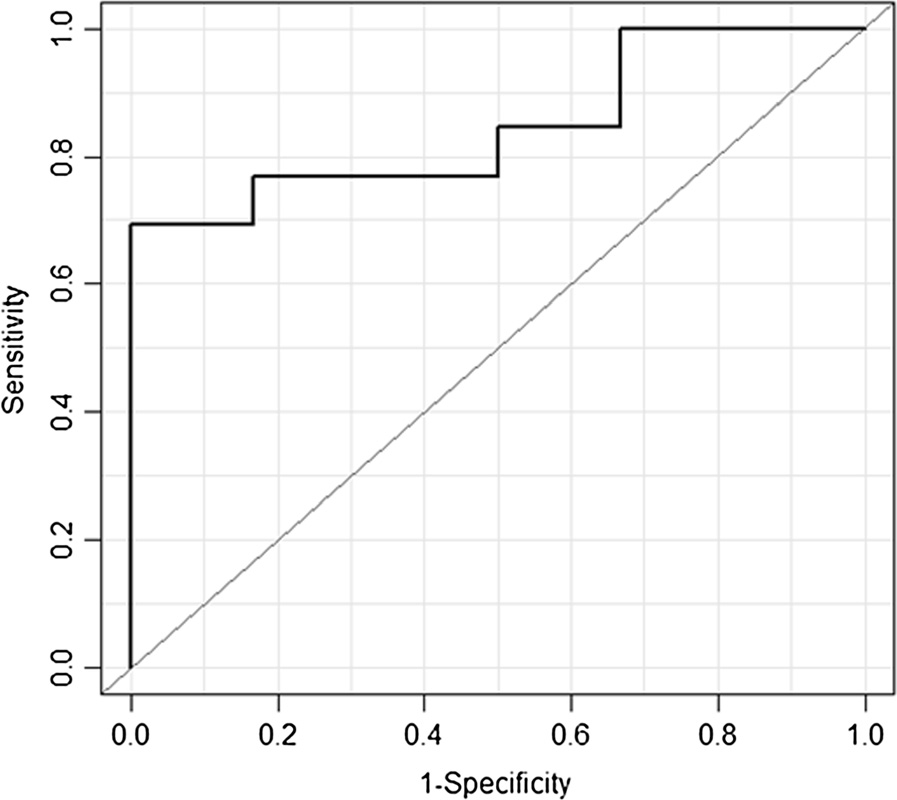
The receiver operating characteristic (ROC) analysis of the eligible electronic medical record (EMR) patient index in 19 trials.

We searched the EMRs for patients who fit the computable criteria characteristics in the 19 trials, counted the number of patients in each trial and calculated the eligible EMR patient index. The accrual achievement demonstrated a significant positive correlation with the eligible EMR patient index (r = 0.67, 95% confidence interval (CI), 0.42 to 0.92; Figure 2). The ROC analysis revealed an estimated 1.7 cut-off value for the eligible EMR patient index, with an area under the curve (AUC) of 0.846, a sensitivity of 69.2% and a specificity of 100.0% (Figure 3). None of the nine trials for which the eligible EMR patient index was less than 1.7 achieved their original target sample size within the scheduled accrual period. There were 16 trials in which 50% or more of trial criteria were matched with the data elements in the EMRs. The relationship of the eligible EMR patient index and the accrual achievement in these 16 trials also exhibited a positive correlation (r = 0.67, 95% CI, 0.38 to 0.96). The ROC analysis revealed an estimated 1.7 cut-off value for the eligible EMR patient index, with an AUC of 0.867, a sensitivity of 70.0% and a specificity of 100.0%.

**Figure 4:**
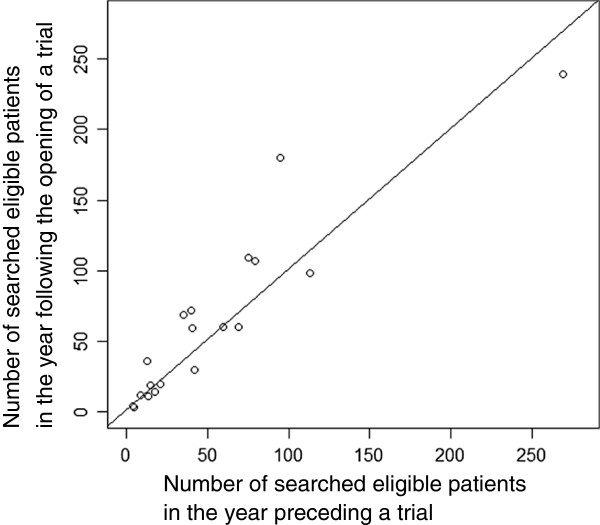
A plot of the number of searched eligible patients both in the year preceding and the year following the opening of a trial.

The number of identified eligible patients in the year preceding the start of the trial was almost consistent with the number of identified eligible patients in the year in which a trial began (Figure 4). The median ratio of the number of eligible patients in the preceding year to the number in the year a trial began was 1.00 (range, 0.36 to 1.67).

## Discussion

We developed a formula to estimate the number of eligible patients using routine EMR data. In half of the tested trials, using the eligible EMR patient index, we were able to predict prior to the start of a trial whether the trial would have a low enrollment because of fewer than expected eligible patients. If researchers are able to accurately predict a shortage of eligible patients, they may modify the eligibility criteria, recruit more participating institutions or abandon the trial to avoid wasting funds and efforts as well as exposing patients to unnecessary risk.

A number of screening methods for EMRs for eligible patients have been developed [7,8,10,11,18-20]. The search method for eligible patients used in our study was based on the patient treatment information rather than the plain text description of the disease in the EMRs. Although we may underestimate the number of potentially eligible patients who were diagnosed with the target disease without standard therapy, we consider those patients to be patients without active disease, patients who are unable to be treated or patients who are unwilling to be treated. Thus, there is little chance to enroll these patients into a clinical trial. When there is no standard therapy for the target disease or the target stage of the disease, one must review the text in the EMRs manually or incorporate an adequate text mining technology to improve the search precision. However, we speculated that the combination of a diagnosis with other information may help refine the estimation [23], and we found that the EMR data and the estimation of the number of patients were accurate enough to predict some of the low enrollment trials.

Approximately one-half of all patient characteristics replaced from the trial eligibility criteria were matched with data elements in EMRs. Considering that the degree of matching in total or by category was not inferior to that achieved in a previous study [21], the included 19 trials are not biased, despite their small number. However, 45% of the patient characteristics were not matched with data elements in the EMRs, which may lead to an overestimation or underestimation of the number of eligible patients in the EMRs. Some trial criteria, such as ‘pregnant or lactating’, ‘measurable disease by RECIST’ and ‘New York Heart Association class I’, were classified as ‘Not applicable’ and were not considered when determining whether the patient should be excluded. In addition, the diagnosis in the EMRs does not necessarily reflect the current condition of the patient. Temporary diagnoses or diagnoses related to the payment of medical insurance are often included in the EMRs and provide false or misleading information that leads to overestimating the number of eligible patients in the EMRs. However, because the exclusion of three trials with low levels of matching did not change the result of the correlation analysis or the ROC analysis, the impact of low levels of matching did not seem to be substantial.

In addition, the eligible EMR patient index is necessary but not sufficient to predict low levels of recruitment. Disappointingly, approximately half (four of ten) of the trials with an eligible EMR patients index greater than 1.7 resulted in low enrollment in this study. This finding results from the index not considering the willingness of patients and from the inaccuracy of EMR data. For instance, only 51% of the eligible patients agreed to participate in a cancer trial at one university-based cancer center [24]. Indeed, the consent rates in the trials conducted in our center ranged from 25% to 100% (data not shown). Four times as many patients as the number of eligible patients in EMRs are necessary when the consent rate of the trial is 25%. Therefore, investigators should consider other disincentive factors that would empirically influence a patient’s consent, such as foreseeable risks or inconveniences to the patients, and the investigators can then determine whether the trial would in fact achieve its target sample size.

Our method also excluded a manual review of the EMRs. In previous studies, the eligible patients were generally identified by the EMRs in two steps: (1) the patient characteristics were screened and matched with standardized codes (for example, ICD codes) or numeric data; and (2) the medical records were manually reconfirmed by the medical staff [10,11]. Our aim was to obtain the total number of eligible patients instead of examining whether an individual patient was eligible for the trial. Therefore, we did not confirm whether the patients searched by the EMR retrieval system were eligible for the trial by verifying the entirety of their EMR data. As a result, the privacy of patients who had not given consent to participate in the trial is protected, while investigators can still speculate on the feasibility of the trial protocol.

The estimated number of eligible patients during the year of the trial accrual period did not exhibit a substantial increase compared to that of the preceding year. Researchers were unable to enroll more patients than they routinely cared for, regardless of the intensity of the recruitment efforts. The acquisition of new patients may be difficult because of the high degree of development among medical institutions and the guaranteed access by Japanese patients to any institution under the comprehensive medical insurance system. Additionally, most patients in Japan would have already been diagnosed or treated by specialists in a branch of medicine [25].

One limitation of this study was that the process of ‘replacement’ was not the exact translation of the trial eligibility criteria into data elements in the EMRs and instead depended on physicians’ conception or the information presented in the trial protocol; thus, disagreement concerning the replacement or incomplete replacement may occur. Another limitation is that the eligible EMR patient index cannot predict all trials that will result in a failure of accrual (sensitivity, 0.645). The index is designed for a single institution and for a relatively small number of target trials. The cut-off value of the eligible EMR patient index in another institution may be different from ours. To speculate whether there are enough potentially eligible patients at a participating trial site for a multicenter clinical trial, each site must be equipped with an efficient EMR retrieval system. Moreover, this study was exploratory in nature, and prospective studies would be needed to validate the predictive ability of the eligible EMR patient index for future clinical trials.

## Conclusions

Our study suggests that in addition to the knowledge of experienced investigators, the health information in EMRs could be a useful component of the feasibility study when planning a clinical trial. Establishing a step to check whether there are likely to be a sufficient number of eligible patients enables sponsors and investigators to concentrate their resources and efforts on more achievable trials.

## Abbreviations

AUC: Area under the curve; EMRs: Electronic medical records; KUH: Kyoto University Hospital; ROC: Receiver operating characteristic; UMIN-CTR: University Hospital Medical Information Network Clinical Trial Registry.

## Competing interests

All authors declare that they have no competing interests.

## Authors' contributions

ES designed the study, replaced the trial eligibility criteria with the EMR data, collected data and wrote the manuscript. KY designed the study, developed the ERS system, wrote computer programs and collected data. ST designed the study and conducted the statistical analysis. MS and KY replaced the trial eligibility criteria with the EMR data. MY is the owner of the ERS systems and supervised the study. All authors read and approved the final manuscript.
